# Mapping CMV-related immune signatures in blood, aorta and perivascular mediastinal adipose tissue

**DOI:** 10.1098/rstb.2024.0401

**Published:** 2025-11-06

**Authors:** Cassia Labeb, Laventa M. Obare, Quanhu Sheng, Xiuqi Zhang, Rama Gangula, Kisyua Nthenge, Samuel S. Bailin, Mona Mashayekhi, Victoria R. Stephens, Lindsey Stolze, Stephen Priest, Jordan C. Malone, Jared M. Oakes, Curtis L. Gabriel, Elizabeth J. Phillips, Spyros A. Kalams, Simon A. Mallal, Sara Gianella, John R. Koethe, Yan Ru Su, Tarek Absi, Celestine N. Wanjalla

**Affiliations:** ^1^ Division of Infectious Diseases, Vanderbilt University Medical Center, Nashville, TN 37232, USA; ^2^ Department of Biostatistics, Vanderbilt University Medical Center, Nashville, TN 37232, USA; ^3^ Division of Diabetes, Endocrinology, and Metabolism, Vanderbilt University Medical Center, Nashville, TN 37232, USA; ^4^ Department of Pathology, Microbiology, and Immunology, Vanderbilt University Medical Center, Nashville, TN 37232, USA; ^5^ Division of Gastroenterology, Vanderbilt University Medical Center, Nashville, TN 37232, USA; ^6^ Institute for Immunology and Infectious Diseases, Murdoch University, Perth, WA, Australia; ^7^ The Tennessee Center for AIDS Research, Nashville, TN, USA; ^8^ Department of Biomedical Informatics, Vanderbilt University Medical Center, Nashville, TN 37232, USA; ^9^ Division of Infectious Diseases, University of California, San Diego, CA, USA; ^10^ Division of Cardiovascular Medicine, Vanderbilt University Medical Center, Nashville, TN 37232, USA; ^11^ Department of Cardiac Surgery, Vanderbilt University Medical Center, Nashville, TN 37232, USA

**Keywords:** cytomegalovirus, ageing, aorta, TEMRA, adipose tissue, T cells

## Abstract

Cytomegalovirus (CMV) establishes lifelong latency and is linked to immunosenescence in older and immunocompromised individuals. We hypothesize that CMV drives systemic and tissue-specific immune changes that may contribute to cardiovascular disease (CVD). Thoracic aorta, blood and perivascular mediastinal adipose tissue from cardiac surgery patients (*n* = 11) were processed within 30–60 min of excision. CMV IgG titres were quantified through ELISA to determine CMV status: CMV(−) (*n* = 4) and CMV(+) (*n* = 7). Immune profiling was performed using flow cytometry and single-cell RNA sequencing. Analyses included MiloR and differential gene expression. Participants (mean age 69.7 ± 8.4 years) were 80% male and 70% Caucasian. CMV(−) and CMV(+) participants had mean IgG titres of 0.038 and 13.55 IU ml^−1^, respectively. CD8^+^ T-cells expressing CD57^+^, GPR56^+^ and CX3CR1^+^ (CGC) were increased in the blood of CMV(+) participants. In the aorta of CMV(+) participants, CD8^+^ T cells and CD4^+^ T cells had decreased HLA-C expression and suppressed interferon-α pathways. In contrast, the TNF-α signalling pathway was increased. CMV infection shapes immune responses and in this pilot, we observed suppression of interferon-α signalling and increased TNF-α-associated pathways in the aorta. Larger studies are needed to define how CMV-driven immune remodelling contributes to CVD.

This article is part of the discussion meeting issue ‘The indirect effects of cytomegalovirus infection: mechanisms and consequences.’

## 1. Introduction

Cytomegalovirus (CMV), a prevalent beta-herpesvirus, establishes lifelong latency after initial acquisition, persisting in various cell types such as monocytes, endothelial cells and stromal cells [1–4]. CMV reactivation occurs intermittently, especially during states of immune suppression [5]. CMV reactivation, often triggered by monocyte differentiation into macrophages, enables viral spread to endothelial and epithelial tissues [5] and activates inflammatory cascades, priming immune responses while sustaining viral persistence. While CMV establishes a reservoir in myeloid lineage cells, it does not enhance the differentiation of monocytes to macrophages [6].

Systemic inflammation induced by CMV, characterized by elevated cytokines such as interleukin (IL)-18, IL-6 and interferon (IFN)-γ, contributes to immune dysregulation and vascular dysfunction [7,8]. Natural killer (NK) cells are critical in early defence against CMV, though CMV evades these responses through mechanisms like viral IL-10 production [9–11]. Toll-like receptor 2 (TLR2) also recognizes CMV glycoproteins, linking innate and adaptive immunity [12,13]. Myeloid cells, mainly monocytes, play key roles in CMV latency and dissemination [6,14].

Emerging research implicates chronic CMV infection in the pathogenesis of cardiovascular disease (CVD). CMV promotes atherosclerosis, myocardial infarction [15,16] and other vascular disorders by directly affecting endothelial cells [17,18]. Such effects occur during viral reactivation, inducing pro-inflammatory and pro-thrombotic pathways and facilitating plaque formation and vascular damage. Monocyte and macrophage activation during CMV reactivation may contribute to foam cell accumulation—a critical step in atherosclerotic plaque development—while inflammatory mediators such as IL-18 and monocyte chemoattract protein-1 (MCP-1) exacerbate vascular dysfunction and immune cell recruitment [7]. Beyond peripheral blood, CMV affects tissue-resident immune cells. CMV reactivation in vascular microenvironments induces inflammatory and fibrotic markers, exacerbating age-related cardiovascular remodelling [19]. Additionally, CMV’s modulation of lipid metabolism and oxidative stress also perpetuates chronic vascular damage [20,21].

For most immunocompetent individuals, CMV remains asymptomatic; however, with time, CMV profoundly affects the immune system by disrupting homeostasis and contributing to ‘immunosenescence’, or a gradual decline in immune competence associated with ageing [2,22]. This process is linked to increased susceptibility to ageing-related disorders, including CVD [22]. A hallmark of CMV-associated immunosenescence is the expansion of terminally differentiated T cells, often linked to chronic inflammation [23–25]. The resulting pro-inflammatory environment, characterized by IL-6, tumour necrosis factor (TNF)-α cytokines and IFN-γ, accelerates ageing and predisposes individuals to vascular pathologies. This phenomenon, termed ‘inflammaging,’ [22] is increasingly recognized as contributing to cardiovascular risk in ageing populations [15,22,26].

CMV employs sophisticated mechanisms to influence innate and adaptive immunity. CD8^+^ T cells are pivotal in controlling CMV replication, with remarkable clonal anti-CMV T-cell receptor (TCR) repertoire stability for years despite evolving memory T-cell differentiation states [27]. CMV-specific CD8^+^ T cells expand disproportionately in people living with HIV (PLWH) and accumulate as highly differentiated memory populations [28–30]. A significant fraction of these are CD45RA-expressing effector memory CD8^+^ T cells (TEMRA), which exhibit potent functional responses upon reactivation [31,32]. Similarly, CMV-specific CD4^+^ T cells suppress CMV lytic replication and the inflation of CD8^+^ T cells [33]. CD27⁻ CD4^+^ TEMRA cells, more prevalent in older adults and certain autoimmune conditions [34,35], are thought to arise through IL-7-mediated homeostatic proliferation. CD4^+^ TEMRA cells are multifunctional but have a limited lifespan after activation. However, studies on CMV-specific TEMRA cells indicate that they persist long term, proliferate in older and younger individuals alike and—in elderly individuals—specifically CD8^+^ CD45RA^+^ cells undergo reduced apoptosis, leading to their accumulation over time [36].

In PLWH, CD4^+^ TEMRA cell inflation is significantly more pronounced, distinguishing it from conditions involving immune suppression, such as organ transplantation [37]. Among PLWH, CD4^+^ TEMRA and CD4^+^ CD28^−^ T cells have also been associated with incident diabetes [38]. CD4^+^ and CD8^+^ TEMRA cells express senescence-associated surface markers and increased inhibitory receptors but exhibit diminished proliferative capacity compared with other memory T-cell subsets. These features are linked to higher mortality and reduced immune competence, particularly in ageing populations [39]. Notably, CD4^+^ and CD8^+^ T cells that express CX3CR1 (a chemokine receptor that traffics cells to inflamed endothelium), GPR56 (a marker of cytotoxic T cells) and CD57 (senescence or late activation) include both T effector memory cells and TEMRA cells [40]. Furthermore, we showed that CX3CR1^+^ GPR56^+^ CD57^+^ (referred to as “CGC^+^”) CD4^+^ and CD8^+^ T cells are cytotoxic [41] and increased in PLWH who also have diabetes [40,41] and subclinical atherosclerosis [42,43].

Beyond T-cell responses, CD4^+^ T cells support B-cell activation and the production of CMV-specific antibodies, neutralizing infection by blocking viral entry and spread [44–48]. Non-neutralizing mechanisms, such as antibody-dependent cellular cytotoxicity and antibody-dependent phagocytosis, enhance protection by recruiting complement and activating innate and adaptive immunity.

Age-related differences in immune profiles between CMV(+) and CMV^−^(–) individuals highlight the virus’s potential impact on immune ageing. While most studies have focused on circulating immune cells, the role of tissue-resident immune cells and non-immune cells across organs remains underexplored. In this exploratory study of a small cohort (*n* = 11), we hypothesize that CMV seropositivity is associated with distinct immune signatures in blood, perivascular mediastinal adipose tissue and the aorta, reflecting systemic and tissue-specific alterations in immune ageing. In this study, we characterize immune responses across these compartments, providing preliminary insights into how CMV may shape immune profiles. These findings are a foundation for future, larger scale studies examining potential links between CMV, immune activation and chronic disease risk.

## 2. Methods

### (a) Clinical cohort

Eleven patients undergoing cardiac surgery for various aortic valve repairs, coronary artery bypass grafting and aneurysm repairs between October 2021 and December 2022 at the Vanderbilt University Medical Center, Nashville, TN consented to the collection of surgically removed tissues (thoracic aorta and perivascular mediastinal adipose tissue) that would otherwise be discarded for research purposes. Tissue samples were collected from the operating room (OR) within 30 min of their excision. Concurrently, blood samples were collected post-resection. Five participants provided matched samples from all three tissues (blood, aorta and adipose). Additionally, five participants provided matched samples from blood and aorta only. One participant provided a sample from the aorta only. Participant metadata collected included age, race and sex. This study was approved by the Vanderbilt University Institutional Review Board.

### (b) Blood and tissue processing

The thoracic aorta and perivascular mediastinal adipose tissue were processed within 30−60 min of surgical excision. Peripheral blood was drawn immediately after the tissues were obtained in the OR. Peripheral blood mononuclear cells (PBMC) were processed using the Ficoll gradient, as previously published [40]. Aorta and perivascular mediastinal tissue (stromal vascular fraction) samples were digested in collagenase I (Roche Catalogue #11088866001, final concentration of 2 mg ml^−1^) overnight at 37°C, 5% CO_2_. The tissue was dissociated using the gentle MACS dissociator (Miltenyi Biotec) and filtered through a 70 µm strainer. The following two washes were performed in PBS and RBC lysis was performed using ACK buffer. The cells were cryopreserved in freezing media (10% dimethyl sulfoxide, 90% foetal bovine serum).

### (c) Cytomegalovirus ELISA

CMV IgG titres in plasma were quantified using a commercial kit (AVIVA Systems Biology, #OKNA00121). Plasma was diluted 1 : 40 and added to the pre-coated ELISA plates in duplicate. After 30 min of incubation, the plate was washed and incubated with enzyme conjugate for 30 min at 37°C. The plate was washed and incubated with 100 μL of TMB (3, 3', 5, 5'-tetramethylbenzidine) substrate for 15 min at 37°C. After adding 100 μL of stop solution (1N HCl), the optical density absorbance was measured at 450 nm within 15 min. The mean intra-assay Coefficient of Variation (CV) across 43 samples measured in duplicate within a single assay run was 3.34%. CMV(+) was defined as titres >1 IU ml^−1^, while CMV(−) was defined as titres below this and aligns with the manufacturer’s lower limit of detection.

### (d) Droplet digital PCR

We used DNA to detect single copies of CMV DNA. The following primers and probes were used for CMV detection: glycoprotein gBFORWARD (FOR) 5′−GAGGACAACGAAATCCTGTTGGGCA−3′, REVERSE (REV) 5′− GTCGACGGTGGAGATACTGCTGAGG−3 and probe 5′−6-FAM− CAATCATGCGTTTGAAGAGGTAGTCCA-BHQ1, US28 FOR 5′−TTTGGTGGATCTTTGCCGTG−3′, REV 5′−ACGAAAGCACCAAGCATGAGTTC−3′ and probe 5′−6-FAM− ATCGCCATTCCACACTTTATGGTGGTG−BHQ1−3′. VIL-10 FOR 5′− TGTTGAGGCGGTATCTGGAGA−3′, REV 5′−CCGTCTTGAGTCCGGGATAG−3′ and probe 5′−6-FAM−TTTCCCGCAGGCGACCACG−BHQ1−3′. CMV detection primers were multiplexed with the RNase P (RPP30) housekeeping gene, as previously described [37]. Positive droplet thresholds were determined using no-template controls. Thermocycling conditions included reverse transcription at 50°C for 60 min, initial denaturation at 95°C for 10 min, 40 cycles of 95°C for 30 s and 60°C for 1 min, with a final enzyme deactivation at 98°C for 10 min.

### (e) Flow cytometry

Cryopreserved PBMC, aorta and perivascular mediastinal adipose tissue samples were thawed and stained with CX3CR1-PE (BioLegend, #149005) and CCR7-BV421 (BioLegend, #353207) at 37°C for 15 min. Cells were then incubated with a multiparameter antibody panel for 20 min at room temperature, enabling comprehensive immune phenotyping. The panel included a live/dead stain (Aqua) to exclude non-viable cells, CD3-BV786 (BioLegend, #565491) to identify T cells, CD4-PcP Cy5.5 (BioLegend, #344607) and CD8-A700 (BD Biosciences, #557945) for delineating helper and cytotoxic Tcells, respectively. CD45RO-PE CF594 (BD Biosciences, #562299) was used to distinguish memory T cells, while CD57-FITC (BioLegend, #359603) marked terminally differentiated effector T cells. GPR56-PECy7 (BioLegend, #358205), a marker of cytotoxic cells, and HLA-DR7-APC Cy7 (BioLegend, #307617) indicated cell activation status. Samples were analysed on a BD FACS Aria II.

### (f) 10× Single-cell transcriptomic analysis

Each participant’s PBMC, aorta and perivascular mediastinal adipose stromal vascular fraction samples were processed in parallel. Cells were stained with unique hashtag antibodies (Total Seq C) and incubated at 4°C for 30 min. The cells were washed in cell staining buffer (PBS with 0.02% bovine serum albumin (BSA)) and stained with total Seq C antibodies (BioLegend: CD163, #333637; CD206, #321147; CD9, #312121; CD56, 305045; CD11b, 301359; CD14, #301859; CX3CR1, #355705; GPR56, #358209; CD16, #302065; CD57, #393321; CD8, #344753; CD3, #300479 and CD4, #344651). For sequencing, cells from different participants were pooled by tissue type and up to 10 000 cells per participant were loaded onto a Chromium Single Cell 5′ assay. Prepared libraries were sequenced on Illumina’s NovaSeq 6000 S2 platform, targeting 50 000 reads per cell for the 5′ assay and 20 000 reads per cell for the Feature Barcode assay.

The raw data were processed using the 10× Genomics Cell Ranger pipeline (v.7.0.1). Then, the cells were demultiplexed by integrating both HTO-based scDemultiplex [49] and mutation-based Souporcell. Filtering retained cells with 200−8000 unique genes, ≥500 read counts and ≤10% mitochondrial content. The individual samples were integrated by FastMNN [50] and clustering analysis was performed through Seurat [51] with a resolution of 0.5. Each cluster was classified as NK/T, fibroblast/smooth muscle cell (SMC), myeloid, B cells, endothelial or platelet based on scMRMA [52], SingleR [53], SignacX [54] and Azimuth and then manually validated by curated marker genes. Sub-clustering was performed on different resolutions and the best resolution was determined by sub-cell type annotations from SingleR, SignacX and Azimuth. For each sub-cell type, Empirical Analysis of Digital Gene Expression in R (edgeR v.4.2.1) [55] was utilized to identify differential expressions across conditions using the glmQLFit function. Gene set enrichment analysis was performed using the gene set enrichment analysis (GSEA) package [56]. MiloDE [57] and MiloR [58] were also used to identify differentially expressed genes and differential cell type abundance in spatial neighbourhoods across conditions.

### (g) Statistical analysis

For flow cytometry data, non-parametric tests were used to assess differences in immune cell proportions between the tissues and participants stratified by CMV plasma titres. The statistical analyses will be structured to account for the partially matched nature of the dataset. The Mann-Whitney U test was used to compare cell proportions between CMV(−) and CMV(+) groups, and Kruskal-Wallis with Dunn’s testing for multiple comparisons was conducted to evaluate relationships between T-cell memory subsets in the aorta, perivascular mediastinal adipose and PBMC. Single-cell RNA-sequencing data were analysed using the MiloR algorithm to identify differentially abundant (DA) cell neighbourhoods and associated transcriptional changes. Uniform manifold approximation and projections for dimension reduction (UMAP) were generated to visualize cell clusters across tissues, stratified by CMV status. MiloR was applied to calculate DA neighbourhoods with a false discovery rate (FDR) to identify statistically significant differences. Differentially expressed (DE) genes in the different neighbourhoods were analysed using MiloDE and volcano plots were generated to depict significant gene expression changes. Statistical significance was determined at an FDR threshold of <0.05. Data visualization included violin plots, UMAP projections, volcano plots and boxplots to illustrate cell type abundance, gene expression variability and neighbourhood-level differences across groups. Analytical tools and parameters (e.g. *k* = 100 for neighbourhood size) were optimized for the reliable detection of differences in cellular and molecular profiles.

## 3. Results

### (a) Participant characteristics

Eleven donors undergoing cardiothoracic surgery consented to this study and were grouped based on plasma CMV IgG titre. The CMV(−) donors (*n* = 4) with CMV titre <1 IU ml^−1^ had a mean age of 65.3 (±9.0 years), while the CMV(+) donors (*n* = 7) had a mean CMV titre of 13.55 IU ml^−1^ and a mean age of 73.2 ± 6.8 (table 1). This pilot cohort was predominantly male (80%) and Caucasian (70%).

**Table 1.** Cohort demographics

	CMV(−) (*n* = 4)	CMV(+) (*n* = 7)	all participants (*n* = 11)
age, years	65.3 [±9.0]	73.2 [±6.8]	69.7 [±8.4]
sex, female	25% [1/4]	17% [1/6]	20% [2/10]
race, White	75% [3/4]	67% [4/6]	70% [7/10)
CMV titre, IU ml^−1^	0.038 [±0.044]	13.55 [±4.90]	8.63 [±7.80]
For age and CMV titre, we report mean [±s.d.] values			

### (b) CGC^+^ CD8^+^ T cells are higher in the blood of donors with high CMV plasma titre

**Figure 1.**
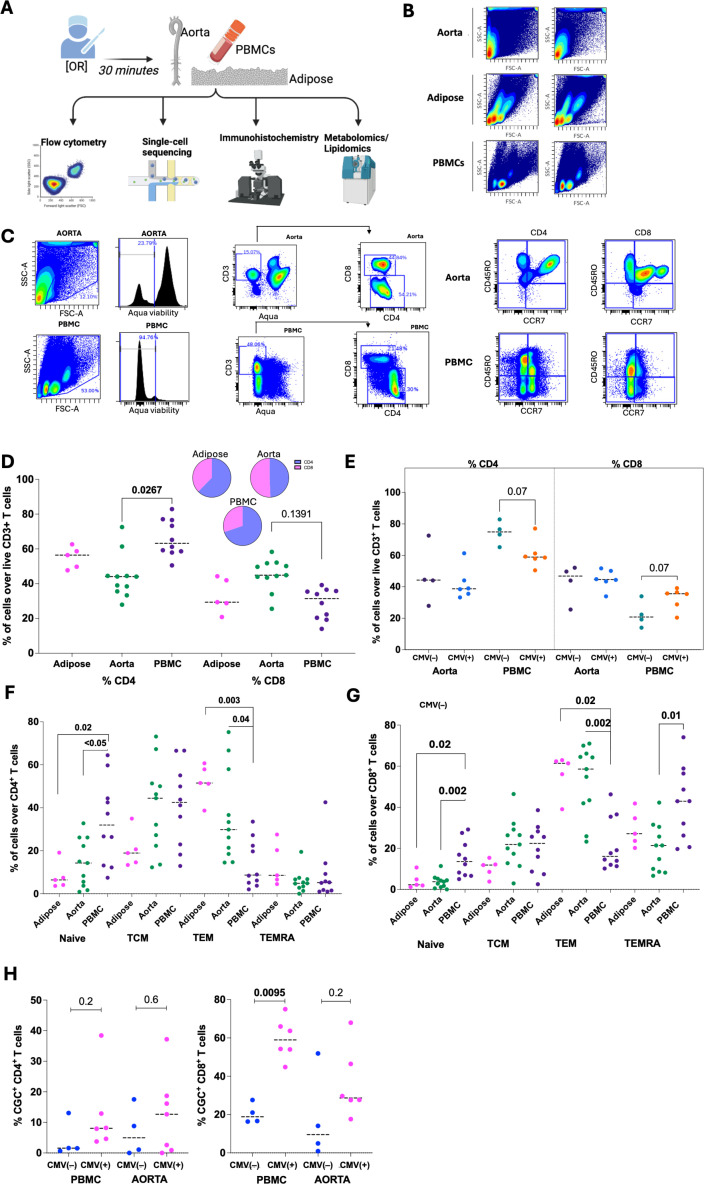
Flow cytometry analysis of T cells in perivascular mediastinal adipose, aorta and PBMC. (A) The workflow shows the tissues and blood obtained and analyses performed.(B) Two-dimensional plots show the FSC-A and SSC-A of cells in the aorta, perivascular mediastinal adipose and PBMCs from a representative sample.(C) Gating of matched aorta and PBMC, starting with a gate that captures cells of interest, followed by a histogram showing live cells, CD4^+^ and CD8^+^ T cells among CD3^+^ T cells and memory T cells (CCR7^+^ CD45RO^−^ naive; CCR7^+^ CD45RO^+^ TCM; CCR7^−^ CD45RO^+^ TEM; CCR7^−^ CD45RO^−^ TEMRA) from one donor. (D,E) Dot plots show the proportion of CD4^+^ and CD8^+^ T cells in the aorta (*n* = 11) and PBMC (*n* = 10) and perivascular mediastinal adipose (*n* = 5). Inset pie charts show CD4/CD8 proportions (D) among all donors and (E) stratified by CMV status .(F,G) Dot plots show naive, TCM, TEM , TEMRA , as well as % CGC^+^ CD4^+^ T cells, and % CGC^+^ CD8^+^ T cells in PBMC and aorta by CMV status. (H). (D,F,G)Statistical analysis includes the Kruskal–Wallis with Dunn’s testing for multiple comparisons and (E,H) the Mann–Whitney *U* test.

The study workflow diagram in figure 1A depicts the different assays performed. Flow cytometry was used to analyse immune cells in the blood (*n* = 10), aorta (*n* = 11) and perivascular mediastinal adipose (*n* = 5) (figure 1B). A representative flow cytometry plot shows the gating of lymphocytes in the aorta and PBMC (figure 1C). In this participant, 12.1% of gated cells in the aorta and 53.0% in PBMC fell within the lymphocyte gate. Lymphocytes were further classified into T cells gated on CD3^+^ cells (CD4^+^ and CD8^+^) that are naive (CCR7^+^ CD45RO^−^), central memory (TCM) (CCR7^+^ CD45RO^+^), effector memory (TEM) (CCR7^−^ CD45RO^+^) and effector memory RA^+^ (TEMRA) (CCR7^−^ CD45RO^−^). A similar gating strategy was used to analyse the perivascular mediastinal adipose.

The aorta had significantly fewer CD4^+^ T cells than PBMC (figure 1D). CMV IgG titres were used as a surrogate marker of prior CMV exposure to stratify participants as CMV(+) or CMV(−) and to explore its influence on T-cell phenotypes. While not statistically significant, CMV(+) donors had 13.7% fewer CD4^+^ T cells (74.5 ± 7.4 versus 60.8 ± 8.8, *p* = 0.07) and 10.3% more CD8^+^ T cells (22.4 ± 8.5 versus 32.7 ± 6.9, *p* = 0.07) in PBMC than in CMV(−) donors (figure 1E). Regarding memory T cells, PBMC were enriched in naive and TCM cells, whereas the aorta and perivascular mediastinal adipose tissue had higher proportions of TEM subsets. CD8^+^ TEMRA cells were significantly higher in PBMC than in the aorta (figure 1F,G). We also analysed the proportion of CGC^+^ (CX3CR1^+^ GPR56^+^ CD57^+^) T cells stratified by the CMV status. There was no difference in CGC^+^ T-cell proportions between PBMC and aorta (electronic supplementary material, figure S1). Additionally, CGC^+^ CD4^+^ T-cell proportions in the aorta and PBMC did not differ between the CMV(−) and CMV(+) donors (figure 1H). However, proportions of CGC^+^ CD8^+^ T cells were significantly higher in the PBMC of CMV(+) donors (20.45 ± 5.3 versus 59.6 ± 10.7, *p* < 0.01).

Like prior studies, our pilot study suggests differences in T-cell subsets owing to CMV status in PBMC, but larger cohorts are needed for tissue aorta and adipose. While these findings suggest an association between CMV status and T-cell phenotypes across different tissues, the mean age of the CMV(+) group was higher than the CMV(−) group (73.2 versus 65.3 years). So age may contribute to the observed differences. In the elderly, CD8^+^ CD45RA^+^ (TEMRA) decay is slower, leading to the accumulation of these cells [36]. Future studies with larger, age-matched cohorts are warranted to confirm these findings.

### (c) Cellular abundance and gene expression differences revealed by MiloR analysis in single-cell RNA-sequence

**Figure 2.**
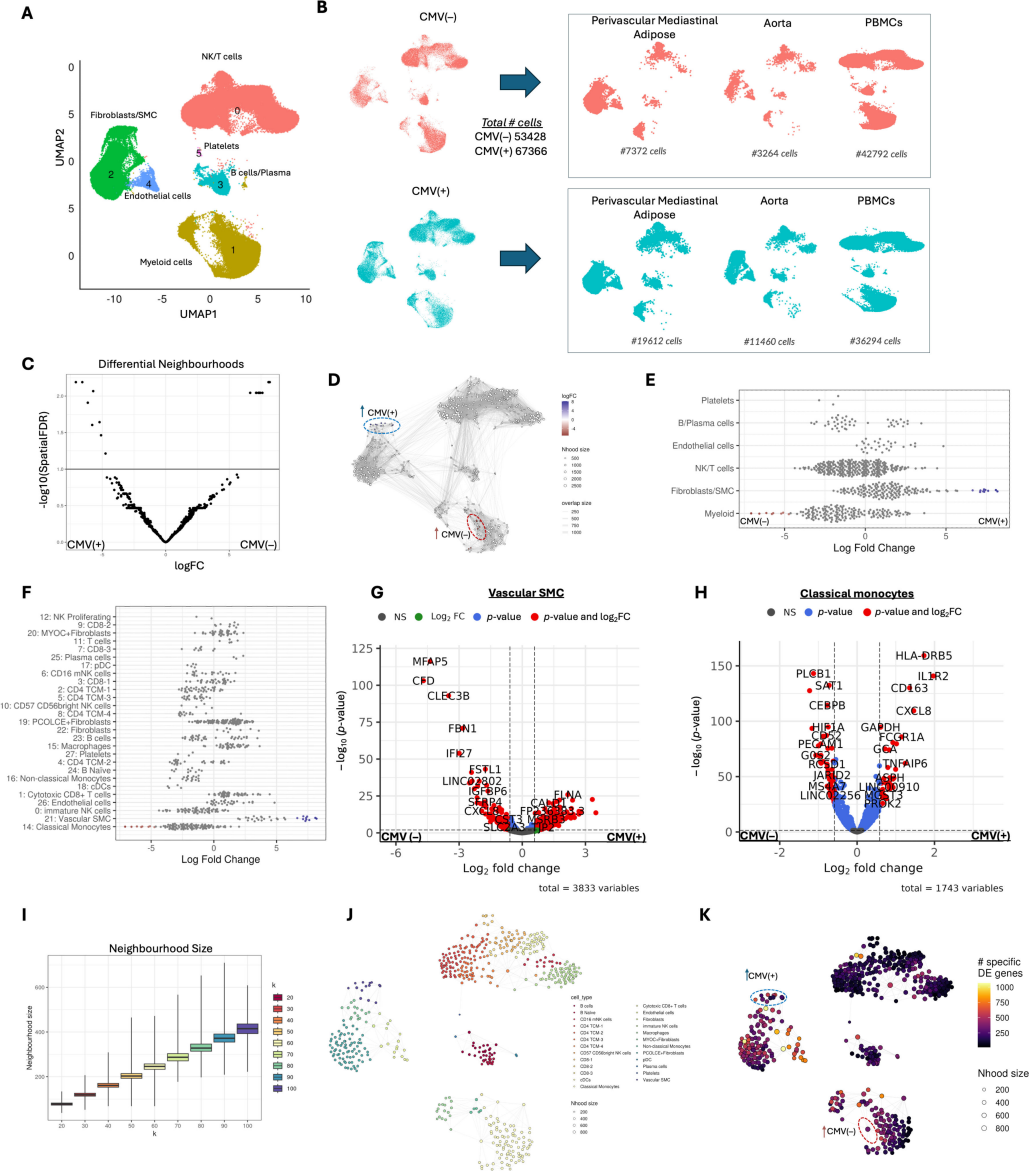
CMV-specific alterations in cellular abundance and gene expression revealed by Milo analysis in single-cell RNA sequence. (A) UMAP plots show single-cell transcriptomic profiles from perivascular mediastinal adipose tissue (*n* = 5), aorta (*n* = 8) and blood (*n* = 7), with clusters of major cell types, including endothelial cells, myeloid cells, NK/T cells, fibroblasts/SMCs, platelets and B cells/plasma cells.(B) UMAPs were stratified by tissue and CMV status, with the number of single cells noted.(C) Volcano plot of differential abundance of MiloR neighbourhoods, with log fold change on the *x*-axis and −log10-adjusted *p*-value on the *y*-axis.(D) A neighbourhood network plot was generated using MiloR. Each node represents a group of cells with similar transcriptomic profiles and edges represent shared cells between neighbourhoods. The position of each node is plotted in the UMAP based on the average coordinates of the cells in that neighbourhood. Nodes are coloured by each neighbourhood’s most abundant assigned cell type.(E) Violin plots show the differential abundance across major cell types; fibroblast/SMC and myeloid neighbourhoods show significant differences in abundance between CMV(+) and CMV(−) groups.(F) A similar plot shows variability in distinct cell subtypes. (G–H) Volcano plots show the differential gene expression (DGE) of VSMC and classical monocytes using edgeR by CMV status. (I) Boxplot shows the *k* (number of nearest neighbours) versus the neighbourhood size;(J) *k* = 100 was picked for an average neighbourhood size of approximately 400 cells. (K) Neighbourhoods are depicted in high-dimensional space, with node size reflecting the neighbourhood size and colour-coded based on dominant cell types. UMAP with an overlying heatmap shows the number of DE genes by MiloDE

Next, we performed single-cell RNA sequencing of PBMC, aorta and perivascular mediastinal adipose. Using MiloR to explore cellular abundance and gene expression changes owing to CMV exposure, we analysed 120 794 cells that passed quality control analysis (from PBMC, aorta and perivascular mediastinal adipose tissue). UMAP visualization revealed clusters of NK/T cells, myeloid cells, fibroblast/smooth muscle cells (SMCs), B cells and platelets (figure 2A). When stratified by CMV, there were 53 428 cells in the CMV(−) and 67 366 from the CMV(+) group and the contributions of perivascular mediastinal adipose, aorta and PBMC are shown on the right (figure 2B).

Differential abundance analysis showed distinct neighbourhoods enriched in CMV(+) (blue) and CMV(−) (red) groups (figure 2C,D). Fibroblast/SMC neighbourhoods were higher in CMV(+), whereas myeloid neighbourhoods predominated in CMV(−) (figure 2E). Sub-clustering identified an increased abundance of vascular smooth muscle cells (VSMCs) in CMV(+) and classical monocytes in CMV(−) (figure 2F). We used edgeR to analyse the differential gene expression (DGE) in all VSMCs (predominantly from the aorta) and classical monocytes (predominantly from PBMC) by CMV status (figure 2G,H). Genes increased in VSMC of CMV(+) participants enriched for myogenesis and mitotic spindle. Genes DE in classical monocytes from CMV(+) participants enriched for oxidative phosphorylation. To investigate cell-type-specific differential abundance between CMV(+) and CMV(−) participants, we applied MiloDE, which performs differential testing across cell neighbourhoods in a *k*-nearest neighbour (kNN) graph. We evaluated neighbourhood size distributions across a range of *k* values (*k* = 20−100) (figure 2I) and selected *k* = 100 for subsequent analysis. In figure 2J, each node represents a neighbourhood of cells coloured by dominant cell type and scaled by neighbourhood size. The neighbourhood graph shows the separated immune and stromal cell types of clusters. Since each cluster predominantly comprises a single cell type, the neighbourhood graph likely captures meaningful biological differences between cell populations. We further visualized the graph by colouring each neighbourhood based on the number of genes significantly DE between CMV(+) and CMV(−) samples (figure 2K). Warmer colours (yellow to red) indicate neighbourhoods with more DE genes, while cooler tones (dark purple) reflect neighbourhoods with few or no DE genes. DGE analyses between CMV(−) and CMV(+) samples were performed across all cells (electronic supplementary material, table S1), as well as in tissue-specific subsets, including PBMC (electronic supplementary material, table S2), aorta (electronic supplementary material, table S3) and perivascular mediastinal adipose tissue (electronic supplementary material, table S4). The top neighbourhoods with the most DE genes included B naive cells, cytotoxic CD8+T cells, classical monocytes, and procollagen C-endopeptidase enhancer (PCOLCE) fibroblasts (electronic supplementary material, figure S2).

### (d) Stratification by cytomegalovirus status suggests a role for IFN-α and TNF-α pathways

**Figure 3.**
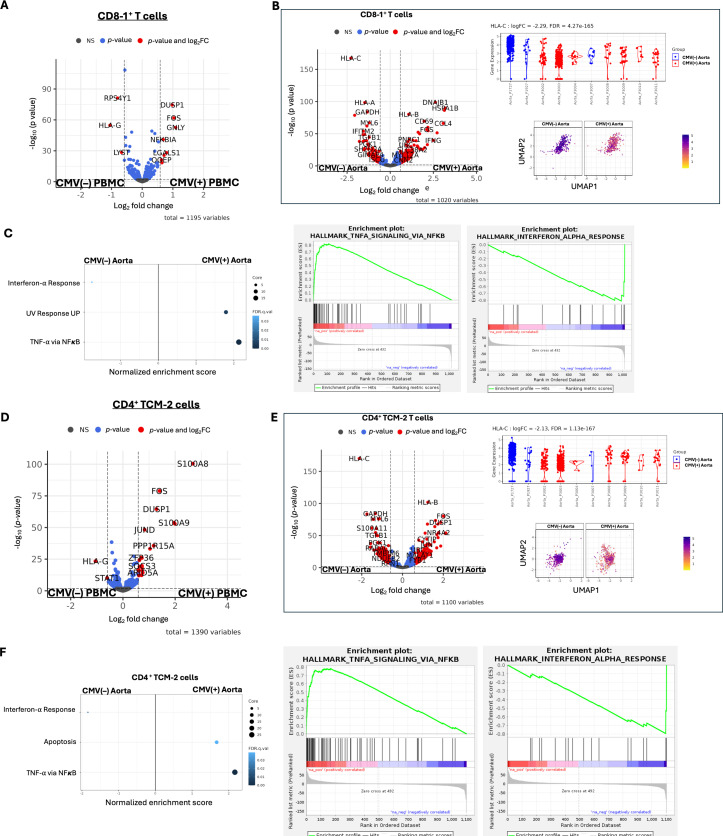
CMV(+) donors have lower HLA-C molecules on CD4^+^ and CD8^+^ T cells in the aorta than CMV(−) donors. (A) The volcano plot shows the DGE of CD8−1^+^ T cells from PBMC by CMV status. (B) Similar analysis of CD8-1^+^ T cells from the aorta by CMV status, with violin plots showing HLA-C expression in individual samples by CMV status. The UMAP shows the differential expression of HLA-C in CD8-1^+^ T cells. The bubble plot shows the pathways over-represented by the genes DE in CD8-1^+^ T cells from the aorta (left) and GSEA plots [56] for the top pathways. The volcano plot shows the DGE of CD4^+^ TCM-2 cells from PBMC by CMV status and the GSEA below (C) shows pathways over-represented. (D) Similar analysis of CD4^+^ TCM-2 cells from the aorta by CMV status, with violin plots showing HLA-C expression in individual samples by CMV status. The UMAP shows the differential expression of HLA-C in CD4^+^ TCM-2 cells. The bubble plot shows the pathways over-represented by the genes DE in CD8-1^+^ T cells from the aorta and GSEA plots for the top pathways.

DGE of all cells by CMV status using edgeR showed differences in cell type and tissue-specific gene expression, with the strongest transcriptional differences observed in NK/T cells and vascular-associated stromal cells (electronic supplementary material, figure S3A–D). T cells from PBMC revealed significantly higher expression of *DUSP1, FOS, GNLY, NFKBIA* and *LGALS1* in CD8^+^ central memory T cells of CMV(+) donors compared with CMV(−) (figure 3A**,** electronic supplementary material, table S5). These genes did not enrich any Hallmark pathways. HLA-G was increased in the CD8^+^ central memory T cells of CMV(−) PBMC. Tissue-specific differences were evident in the aorta, where CD8^+^ central memory T cells from CMV(+) donors had decreased HLA-C, HLA-A and TGFβ expression compared with CD8^+^ central memory T cells from CMV(−) donors (figure 3B, electronic supplementary material, table S6). The pathway enrichment analysis revealed enhanced TNF-α/NFκB signalling in CD8^+^ T cells from CMV(+) and suppression of the IFN-α pathway (figure 3B, electronic supplementary material, tables S7-S8). CD4^+^ TCM-2 cells from the PBMC of CMV(+) donors showed increased expression of genes that enriched in TNF-α/NFκB signalling compared with CMV(−) donors (figure 3C, electronic supplementary material, tables S9-S10). CD4^+^ TCM−2 cells from the aorta also had lower HLA-C expression in CMV(+) donors but higher *HLA-B* (figure 3D, electronic supplementary material, table S11) and increased TNF-α signalling via the NFκB and suppression of the IFN-α pathway (electronic supplementary material, table S12).

In perivascular mediastinal adipose tissue, DGE analysis of CD8^+^ T cells and CD4^+^ TCM-2 cells by CMV titre groups did not identify significant gene expression differences. However, in CMV(−) donors, T cells from the aorta showed significantly higher *HLA-C* expression than T cells from perivascular mediastinal adipose tissue (electronic supplementary material, figure S4A–D, tables S13–S14). A similar analysis in CMV(+) donors, comparing aorta and adipose, did not show differences in *HLA-C* (electronic supplementary material, figure S5A,B, tables S15–S16). In VSMC from the aorta, CMV(+) showed decreased IFN-γ and IFN-α responses compared with CMV(−) (electronic supplementary material, figure S6A-C, tables S17–S19). PCOLCE fibroblasts from CMV(+) versus CMV (−) donors also demonstrated suppression of the IFN-α response pathway (electronic supplementary material, figure S6D–F, tables S20,S21).

DGE analysis of endothelial cells in the perivascular mediastinal adipose by CMV status showed that genes higher in CMV(−) donors enriched for the TNF-α signalling pathway and mechanistic target of rapamycin complex 1 (MTORC1) compared with CMV(+) (electronic supplementary material, figure S7A-C, tables S22-S23). CMV(+) donor endothelial cells in aorta upregulated genes enriched for oxidative phosphorylation (electronic supplementary material, figure S7D–F, tables S24–S25).

## 4. Discussion

This study provides insights into the influence of CMV on immune and non-immune functions in the context of cardiovascular disease, leveraging tissue and blood from participants with a history of coronary artery disease or aneurysms undergoing cardiothoracic surgery. We identified distinct immune profiles in each compartment by comparing T-cell surface markers and transcriptomic profiles across the aorta, perivascular mediastinal adipose tissue and peripheral blood. The aorta contained an approximately equal distribution of CD4^+^ and CD8^+^ T cells, while blood and perivascular mediastinal adipose tissue were enriched in CD4^+^ T cells. TEMs predominated in the aorta and perivascular mediastinal adipose tissue. In contrast, blood showed higher proportions of naive T cells and CD8^+^ TEMRA cells, consistent with the established roles of these subsets.

### (a) CMV and immune function

CMV(+) donors had an increased proportion of cytotoxic CD8^+^ T cells in PBMC, with a similar trend in the aorta, though not statistically significant. Our results are similar to prior studies where CMV reactivation promotes the accumulation of senescent and cytotoxic T cells in the blood, contributing to systemic inflammation and immune exhaustion [26,31]. Transcriptional analysis revealed distinct expression profiles in T cells stratified by CMV status. *Fos,* genes associated with inflammation and cell activation [59] were increased in T cells from CMV(+) donors.

Pathway enrichment analysis revealed that TNF-α signalling was predominant and the IFN-α pathway was suppressed in T cells from the aorta of CMV(+) individuals. Additionally, *HLA-C* transcripts, critical for anti-CMV responses [60], were more highly expressed in T cells from CMV(−) individuals than in CMV(+) individuals, suggesting a potential impairment in antigen presentation or immune recognition in the vascular compartment of CMV(+) donors. HLA-C is a class I MHC molecule primarily involved in antigen presentation to CD8^+^ T cells. Still, its expression in CD4^+^ T cells may have an indirect role in shaping immune responses. One possible mechanism is that CD4^+^ T cells, through cytokine production or cell–cell interactions, help shape NK cell function. HLA-C is a key ligand for killer cell immunoglobulin-like receptors (KIRs) on NK cells, which regulate NK cell activation and cytotoxicity. Lower HLA-C expression in CD4^+^ T cells could contribute to less effective NK cell licensing or activation, diminishing anti-CMV responses and allowing the virus to persist. CD4^+^ T cells are also central in coordinating immune responses, supporting CD8^+^ T-cell function and maintaining a balanced inflammatory environment. The decreased *HLA-C* expression in CD4^+^ T cells of CMV(+) donors could reflect an immune state that does not fully support effective antiviral responses. TNF-α signalling via NFκB, enriched in CD8^+^ and CD4^+^ TCM-2 cells from CMV(+) donors, is consistent with a heightened inflammatory response that may contribute to immune exhaustion. In contrast, CMV(−) donors exhibited localized TNF-α signalling enrichment primarily in stromal and endothelial cells, which may reflect tissue-specific immune surveillance or a homeostatic signalling role rather than chronic activation [61].

### (b) CMV and tissue-specific immune responses

CMV has been implicated in homeostatic and pathogenic immune cell accumulation across tissues. The heterogeneity of CMV’s impact across tissues is underscored by a prior study using the genotype-tissue expression (GTEx) project, which identified varying CMV viral loads in different tissues, including high levels in adipose, colon and muscle, and lower CMV transcripts in blood vessels [62]. These differences may explain the role of tissue-specific factors in shaping immune responses and inflammation associated with CMV. In a recent study, whole virome profiling revealed that CMV was the only virus associated with the accumulation of these senescent CD8^+^ T cells and that a higher proportion of circulating CMV-specific senescent CD8^+^ T cells, rather than CMV-seropositivity alone, was associated with chemotherapy-induced senescence and poorer outcomes in advanced non-small cell lung cancer [63].

In our study, which included participants with a mean age of 69.7 years and varying CMV titres, we observed a significantly higher proportion of CGC^+^ CD8^+^ T cells in PBMC from CMV(+) donors, even with a sample size of 11 participants. Notably, CGC^+^ CD8^+^ T cells are both senescent and cytotoxic [41,43]. However, this relationship was not observed in the aorta or perivascular mediastinal adipose tissue, which may be attributable to limited sample size or may instead reflect differences in the tissue-specific microenvironment that influence immune cell recruitment, retention and survival [64]. Notably, we did not detect CMV transcripts in the aorta and adipose tissue (electronic supplementary material, figure S8), which may suggest that the recruitment of CMV-specific cells during chronic infection is likely mediated by the recruitment of cells into tissues in a stochastic manner through chemokine receptors, such as CX3CR1 binding to inflamed endothelium. However, a larger cohort is needed to conclude this.

### (c) Broader context and adaptive immune variability

Latent CMV infection profoundly influences adaptive immune cell variation, as demonstrated in a large study of 1000 healthy individuals of Western European ancestry. CMV seropositivity emerged as a dominant factor affecting adaptive immunity. In contrast, non-HLA genetics mainly shaped the innate immune system [65]. Other factors like smoking, age and sex were comparatively less influential. Our cohort of ageing participants with cardiovascular disease aligns with previous findings. It highlights the nuanced interplay between CMV status, immune responses and possible tissue-specific adaptations.

### (d) Mechanisms and implications for cardiovascular disease

**Figure 4.**
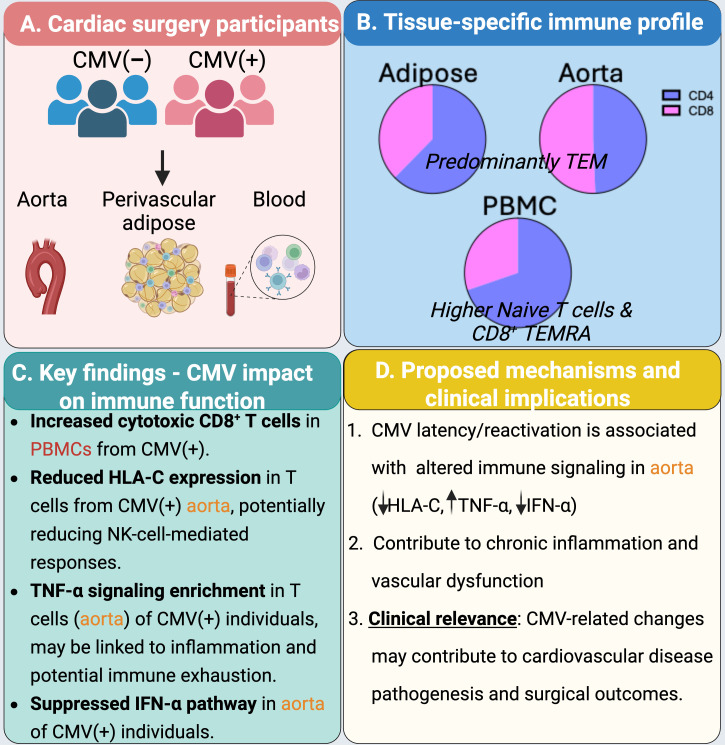
Working model: CMV alters tissue-specific immunity in cardiac surgery patients independent of local viral detection. Samples from cardiac surgery participants with or without CMV infection were analysed for immune profiles in aortic, perivascular adipose and peripheral blood tissues. Notably, CMV DNA was not detected in the aorta by droplet digital PCR (ddPCR), indicating that direct viral presence is not necessary for CMV-associated immune alterations. Immune composition varied by tissue: adipose and aorta predominantly contained effector memory (TEM) T cells, while peripheral blood showed increased proportions of naive T cells and CD8^+^ TEMRA subsets. Key CMV-driven immune changes included elevated cytotoxic CD8^+^ T cells, reduced aortic HLA-C expression, enhanced inflammatory (TNF-α) signalling and suppressed IFN-α signalling, specifically in the aorta. The mechanisms underlying these tissue-specific immune responses, particularly in the absence of detectable viral DNA in vascular tissue, remain unclear and merit further investigation. These findings suggest that CMV may contribute indirectly to vascular inflammation and dysfunction, impacting cardiovascular disease pathogenesis and clinical outcomes.

The differential expression of *HLA-C* and enrichment of TNF-α and IFN-α signalling pathways suggest key mechanisms by which latent CMV infection alters immune function across tissues. HLA-C-restricted T-cell responses, known to be more robust with ageing and less prone to exhaustion, may be especially important in vascular tissues where chronic inflammation and metabolic stress drive disease [60]. In CMV(+) individuals, PBMC exhibit increased cytotoxic CD8^+^ T cells and CD8^+^ TEMRA cells. In contrast, aortic T cells show reduced HLA-C expression, heightened TNF-α signalling and suppressed IFN-α pathways—hallmarks of chronic inflammation and immune exhaustion. These CMV-associated immune alterations may impair antiviral surveillance, promote vascular dysfunction and contribute to cardiovascular disease progression and adverse surgical outcomes (figure 4).

### (e) Study limitations

This pilot study is limited by a small sample size (*n* = 11), which restricts statistical power and generalizability and needs validation in a larger cohort. For example, we could not adjust for differences in age between the CMV(−) and CMV(+) participants. Second, the categorization of participants as CMV(−) and CMV(+) was based on plasma IgG titres and while undetectable or very low IgG titres suggest the lack of a substantial humoral immune response to CMV, it does not preclude an exposure to CMV virus in the past or individuals with an inability to mount an antibody response. Third, the cross-sectional design precludes us from drawing causal inferences about the relationship between CMV titres, immune profiles and cardiovascular disease. Fourth, the study population comprised older adults undergoing cardiac surgery, which may not represent broader populations, including younger or healthier individuals. Fifth, blood was collected from the participants during surgery after tissue was resected to match the tissue collected. The transcriptomic profiles may be affected by inflammatory changes related to the surgery. Sixth, tissue-specific microenvironments may introduce variability in immune profiles and our findings may be influenced by sampling or processing biases. Finally, further functional assays are needed to confirm the biological significance of the observed gene expression patterns.

### (f) Future directions

Future studies with a larger cohort of surgical patients undergoing coronary artery bypass surgery or aneurysm repair are underway to validate and improve the generalizability of these findings across different populations. We will also directly measure CMV cellular responses using overlapping CMV peptides incubated with cells from all three compartments. Furthermore, longitudinal studies would provide temporal dynamics of CMV reactivation, immune cell profiles and their relationship to cardiovascular outcomes. Additionally, multi-omics approaches integrating transcriptomics, proteomics and metabolomics could uncover novel biomarkers and mechanistic pathways in systemic and tissue-specific contexts. Finally, linking immune profiles to clinical outcomes, such as recovery from surgery or cardiovascular event rates, could inform the development of CMV-targeted therapeutic interventions to reduce or counter immune dysregulation and chronic inflammation in ageing populations.

## 5. Conclusion

CMV may be associated with distinct transcriptional adaptations that vary by cell and tissue type.

## Data Availability

Supportive data for this manuscript is available in supplementary material [66]. Sequencing data is deposited in NIH Geo with accession number GSE289253.
